# Lysozyme enhances the bactericidal effect of BP100 peptide against *Erwinia amylovora*, the causal agent of fire blight of rosaceous plants

**DOI:** 10.1186/s12866-017-0957-y

**Published:** 2017-02-17

**Authors:** Jordi Cabrefiga, Emilio Montesinos

**Affiliations:** Institute of Food and Agricultural Technology-CIDSAV-XaRTA, University of Girona, Girona, 17003 Spain

**Keywords:** BP100, Antimicrobial-peptide, *Erwinia amylovora*, Fire blight, Lysozyme, Membrane permeabilization

## Abstract

**Background:**

Fire blight is an important disease affecting rosaceous plants. The causal agent is the bacteria *Erwinia amylovora* which is poorly controlled with the use of conventional bactericides and biopesticides. Antimicrobial peptides (AMPs) have been proposed as a new compounds suitable for plant disease control. BP100, a synthetic linear undecapeptide (KKLFKKILKYL-NH_2_), has been reported to be effective against *E. amylovora* infections. Moreover, BP100 showed bacteriolytic activity, moderate susceptibility to protease degradation and low toxicity. However, the peptide concentration required for an effective control of infections *in planta* is too high due to some inactivation by tissue components. This is a limitation beause of the high cost of synthesis of this compound. We expected that the combination of BP100 with lysozyme may produce a synergistic effect, enhancing its activity and reducing the effective concentration needed for fire blight control.

**Results:**

The combination of a synhetic multifunctional undecapeptide (BP100) with lysozyme produces a synergistic effect. We showed a significant increase of the antimicrobial activity against *E. amylovora* that was associated to the increase of cell membrane damage and to the reduction of cell metabolism. Combination of BP100 with lysozyme reduced the time required to achieve cell death and the minimal inhibitory concentration (MIC), and increased the activity of BP100 in the presence of leaf extracts even when the peptide was applied at low doses. The results obtained in vitro were confirmed in leaf infection bioassays.

**Conclusions:**

The combination of BP100 with lysozyme showed synergism on the bactericidal activity against *E. amylovora* and provide the basis for developing better formulations of antibacterial peptides for plant protection.

## Background

Fire blight is an economically important disease affecting rosaceous plants. The causal agent is *Erwinia amylovora* that infects mainly apple and pear as well as a broad range of woody ornamental plants. Control of disease is conducted by an integrated management based on the treatment with antibiotics or copper derivatives combined with the use of appropriate cultural measures [1, 2] and with biocontrol agents [3–5]. However, development of new active compounds with low phytotoxicity, reduced environmental impact and broad spectrum of activity is still required.

Antimicrobial peptides (AMPs) appear as a new class of compounds for plant disease control [6, 7]. AMPs are found in many organisms, including plants, insects, amphibians and humans and are components of the innate immune system. Moreover, some AMPs have been related to antibiosis in microorganisms [7–11]. Most AMPs are small and cationic peptides with the capactiy to adopt an amphipathic conformation [12, 13]. Their antimicrobial activity has been extensively related with the capacity to interact with the cell membranes [14]. This mode of action confers a broad spectrum of action, mainly against bacteria and fungi, and allows the penetration of peptide into the cell, favouring other mechanisms of action targeting nucleic acids and proteins [15–20].

The activity of antimicrobial peptides against pathogen infections in plants, including postharvest products, have been reported [21–24]. As a result of our research focused on the development of new antimicrobial agents, linear undecapeptides (CECMEL11) were disgned using a combinatorial approach. Screening of the CECMEL11 library allow us to identify peptides with activity against several plant pathogenic bacteria including *Erwinia amylovora*, *Pseudomonas syringae* pv. syringae, *Xanthomonas axonopodis* pv. vesicatoria and fungi like *Penicillium expansum* [23, 25, 26]. Peptide BP100, KKLFKKILKYL-NH_2_, was the most active against bacteria and was effective to inhibit infections caused by *E. amylovora* in apple and pear flowers. However, peptide concentrations necessary for the control of fire blight disease were 10 to 50 times higher than the minimal inhibitory concentration (MIC) [26]. This decrease in the activity *in planta* has been attributed to the inactivation by certain plant compounds or structures, or to their degradation by proteases from plant tissues or epiphytic microorganisms [27, 28].

Control of plant diseases through the use of antimicrobial peptides is a potential complement or alternative to conventional bactericides, offering more selective and environmentally friendly products. However, the concentrations of these peptides required to control pathogen infections are generally too high due to the loss of activity when interact with non-target plant compounds or structures, or due to their degradation [26–28]. To improve their stability, several strategies have been used in different peptides and the most common has been to substitute certain aminoacid residues with non-natural aminoacids [29, 30]. In the case of BP100, the incorporation of D-amino acids has increased their stability to protease degradation and its activity in ex vivo and *in planta* assays [31].

However, the improvement of the activity of BP100 through its co-formulation with enhancer compounds (e.g. lysozyme) seems a reliable strategy. Thus, combination of nisin with lysozyme has shown a synergistic effect against Gram positive bacteria [32–34] and lysozyme has been successfully used as an enhancer of the activity of GMAP2 against Gram negative bacteria [35]. For this reason, the use of lysozyme as an enhancer of BP100 activity could be a good strategy because the main mechanism of action of BP100 seems to be associated to the disruption of cell membrane [36, 37].

More in detail, to increase the activity of peptides, different approaches have been suggested such as the combination of the peptide with divalent metal cations [38], with an enzyme [39] or mixtures of peptides with diferent mode of action [40]. Interestingly the combination of nisin (a bacteriocin peptide) with lysozyme improved the effect against *Listeria monocytogenes* [41] and *Clostridium difficile* [32]. It has been described also that the combination of lysozyme with the anionic peptide GMAP2 showed a syngergistic effect on the activity against *Escherichia coli, Klebsiella pneumoniae* and *Pseudomonas aeruginosa* [35]. This synergic effect has been explained by the fact that lyzozyme hidrolyses ß1-4 bonds between N-acetylglucosamine and N-acetylmuramic acid damaging the bacterial peptidoglycane cell wall which subsequently may facilitate cell membrane disruption by the AMP [42]. However, the improvement of the antibacterial activity has not been previously shown for plant pathogenic bacteria, nor with the multifunctional synthetic peptide BP100.

In the present work, the effect of the combination of the multifunctional undecapeptide BP100 with lysozyme against *E. amylovora* has been studied. We analyzed the effect of the combination of BP100 and lysozyme at different doses in the activity against *E. amylovora* in vitro by following the integrity of cell membrane and cell viability. Once optimized the dose combination, the effect of interfering plant extracts was evaluated. Finally, the synergistic effect of BP100 and lysozyme in the inhibition of infections caused by *E. amylovora* was confirmed in wounded pear leaves.

## Methods

### Peptide synthesis

Peptide BP100 (KKLFKKILKYL-NH_2_) was synthesized in the Laboratory of Organic Chemistry of the University of Girona (LIPPSO) by means of solid-phase synthesis [26]. BP100 purity was >95% and was determined by high-performance liquid chromatography (HPLC; Dionex). Peptide identity was confirmed by electrospray ionization mass spectrometry (ESI-MS; Bruker Daltonics) and matrix-assisted laser desorption ionization-time-of-flight (MALDI-TOF; Bruker) analysis. Before use, lyophilized peptide BP100 was dissolved in distilled water to a concentration of 1 mM and sterilized by filtration through a 0.22-μm pore-size filter.

### Bacterial strains and growth conditions

The non-pathogenic *E. amylovora* PMV6076 [43] was used for the analysis of the antibacterial activity in vitro of BP100 peptide and the combination with lysozyme. *E. amylovora* EPS101 was the strain used for the infection assays [44]. Strains were grown overnight at 25 °C in Lysogeny Broth agar (LB) (10 g NaCl, 5 g yeast extract, 10 g triptone, pH 7.4). Fresh colonies grown during 24 h were scraped from the petri dish and suspended in distilled water previously sterilized. Suitable infections in incoulated leaves are achieved only with fresh plate cultures [45, 46]. Cell suspensions were adjusted to 10^8^ CFU/ml on basis of the optical density (OD) and serially diluted in sterile distilled water to get the appropriate concentration for the experiments.

### Viable cell counting

For the viable cells assessment, a method based on the inoculation of the cell suspension in LB broth and monitoring the growth curve was used [47]. The authors demonstrated that several growth curve parameters, like the optical density (OD) at a given time, are directly related to the initial cell concentration. This method was set-up for *E. amylovora* in the present work. Several growth curves using OD were made departing from known initial cell concentrations determined by plate counting. The calibration curve consisted of suspensions of *E. amylovora* PMV6076 at concentrations from 10^2^ to 10^8^ CFU/ml (confirmed by plate counting). Briefly, 20 μl samples (threee replicates for each treatment) were mixed with 180 μl of LB medium in a microplate well and plates were incubated in the multimode reader with 20 s of shaking during 20 h at 25 °C. OD was measured each hour at 600 nm. A relationship between the initial cell concentration by plate counting and the optical density of the growth curve at 20 h was observed (y = 17.902 · x + 8.814; *R* ^*2*^ = 0.97). This relationship was used to estimate the initial cell concentration of samples during the experiments.

### Flow cytometry analysis of cell membrane damage

To determine the effect of BP100 on cell membrane, a SYTOX green based assay was used. The dye only can penetrate cells with their membrane damaged and subsequently binds DNA producing fluorescence upon excitation with UV light [48]. An aliquot of 160 μl of a bacterial suspension of *E. amylovora* PMV6076 adjusted to 10^8^ CFU/ml were mixed with 20 μl of BP100 (15 μM) and 20 μl of SYTOX (5 μM). The cell suspension was incubated during 3 h at 25 °C. A control consisting of 20 μl of water was included. Then, bacterial suspensions were 100 fold diluted in distilled water and analyzed in a flow cytometer (Sony SH800, Sony Biotechnology Inc. IL, USA). At least 10^4^ events per sample were measured. Data were analyzed using SH800 software and scattergrams were generated by combining forward scatter channel (FSC) with SYTOX green fluorescence (Dichroic/Splitter, dichroic long-pass: 550 nm, band-pass filter: 525 nm, detection width 505 to 545 nm).

### Bactericidal activity of BP100 combined with lysozyme

The antimicrobial activity of BP100 was determined at low concentrations (1.0 and 2.5 μM) which are around the MIC [26], or combined with lysozyme (Sigma Aldrich, USA) at 0.125, 0.250 and 0.5 mg/ml. The assay was carried out to determine the membrane damage by SYTOX fluorescence and viable cells, simultaneously. For the membrane permeabilization analysis, 160 μl of a suspension of *E. amylovora* PMV6076 adjusted to 10^8^ CFU/ml were mixed with 20 μl SYTOX Green (Life Technologies, Invitrogen, Madrid, Spain) at 5 μM and 20 μl of the corresponding treatment (BP100, lysozyme). Controls using water instead of the peptide were included. Incubation was performed for 3 h at 25 °C in an automatic spectral scanning multimode reader (Varioskan, Ascent FL; Labsystems, Finland). Uptake of Sytox Green was determined fluorometrically by measurement at 580 nm after an excitation at 495 nm. Samples were taken at given times of each mix treatment (BP100, lysozyme) for viable cells concentration analysis according to the growth curve method described above.

In parallel, the samples were viewed with an optical microscope (NIKON Eclipse Ci-L, NIKON, Germany) with phase-contrast for total bacteria and blue excitation light (Nikon B-2A, excitation 450–490 nm, dichroic mirror 505 nm, longpass > 520 nm) for SYTOX fluorescence stained cells. The images were captured with CCD camera NIKON Digital Sight DS-Fi2 (NIKON, Germany) using the digital image analysis software NIS elements v 3.22 (NIKON, Germany).

### Dose-efect of BP100 alone or combined with lysozyme

The bactericidal activity of BP100 at increasing concentrations (0, 1.5, 3.0, 5.0 and 10 μM) alone or combined with lysozyme (0.05 and 0.5 mg/ml) against *E. amylovora* was determined by measuring membrane damage, metabolic activity and survival of cells. In addition to SYTOX as an indicator of cell membrane damage, resazurin was used as a metabolic activity indicator during the assay [49]. Briefly, 140 μl of a suspension of *E. amylovora* PMV6076 (1 × 10^8^ CFU/ml), 20 μl SYTOX Green at 5 μM, 20 μl of resazurin at 100 μM and 20 μl of the corresponding treatment (BP100 or lysozyme ten fold concentrated). Incubation was performed during 3 h at 25 °C in the scanning multimode reader. Membrane permeabilization and viable cells were determined as previously described, while metabolic activity was assessed with the resazurin reduction to resofurin by fluorometric measurement of the emitted fluorescence at 595 nm after an excitation at 535 nm. Controls using water instead of the peptide were included. Three replicates for each treatment were done.

### Effect of plant extracts on the activity of peptide BP100 combined with lysozyme

The activity of BP100 alone or combined with lysozyme (0.5 mg/ml) was evaluated in plant extracts consisting of young pear leaves (*Pyrus communis* cv. Conference). A leaf sample of 1 g was homogenized in 20 mL of 0.05 M phosphate buffer (pH 7.0) using a stomacher (Masticator, IUL Instruments, UK). The extract was then diluted to 10% in the same buffer, filtered through several layers of cheesecloth and sterilized by filtration through a filter of a pore size of 0.20 μm. Then, a contact test was performed by mixing 800 μl of the plant extract with 100 μl of an *E. amylovora* suspension at 10^7^ CFU/ml and 100 μl of each treatment (BP100, lysozyme). Water was used as non-treated control instead of plant extracts. After an incubation of 3 h at 25 °C, the effect of each treatment was determined as the remaining viable cells using the same methodology previously described. Reduction of viable cells respect to the non-treated control was calculated in order to determine the inhibitory effect of leaf extracts on the activity of the treatments. Three replicates for each treatment were done.

### Leaf infection assays

The efficacy of BP100 alone (25 and 100 μM) or combined with lysozyme (0.5 mg/ml) was determined in the inhibition of infections by *E. amylovora* in detached pear leaves (*P. communis* cv. Conference). The youngest leaves were collected from plants cultivated in the greenhouse. Leaves were surface disinfected for 1 min by immersion in a solution of sodium hypochlorite (1% active chlorine). Then leaves were washed twice in distilled water, and left dry under airflow in a sterile cabinet. Woundes on the leaves were performed by a double incision (∼1 mm) perpendicular to the midrib. Leaves were placed over a humidified paper towel inside plastic boxes. Then, 10 μl of the corresponding treatment were placed onto each wound, and the treated leaves were left for 1 h at room temperature. Then, *E. amylovora* EPS101 was inoculated in the woundswith 10 μl of a suspension adjusted at 10^7^ CFU/ml. Leaves were incubated during 5 days at 23 °C and high relative humidity. Three replicates of of nine leaves per each treatment were performed. A control inoculated with water, a control treated with lysozyme at 0.5 mg/ml, and a reference control treated with streptomycin at 100 μg/ml were included. The intensity of infections was evaluated for each leaf using a severity scale from 0 to 3: 0, no symptoms observed; 1, necrosis located around the wound; 2, necrosis progress far from the wound; and 3, necrosis of whole leaf [37, 46]. The severity was calculated for each replicate. Four independent assays were done.

### Statistical analysis

Analysis of variance (ANOVA) was used to determine the effect of the treatments on the activity against *E. amylovora* on basis of the general linear model (GLM) procedure of Statistical Analysis System (SAS) program (version 8.2; SAS Institute Inc., NC). Tukey’s test was used to separate the means (*P* < 0.05).

## Results

### Cell membrane damage after exposure to BP100

**Fig. 1:**
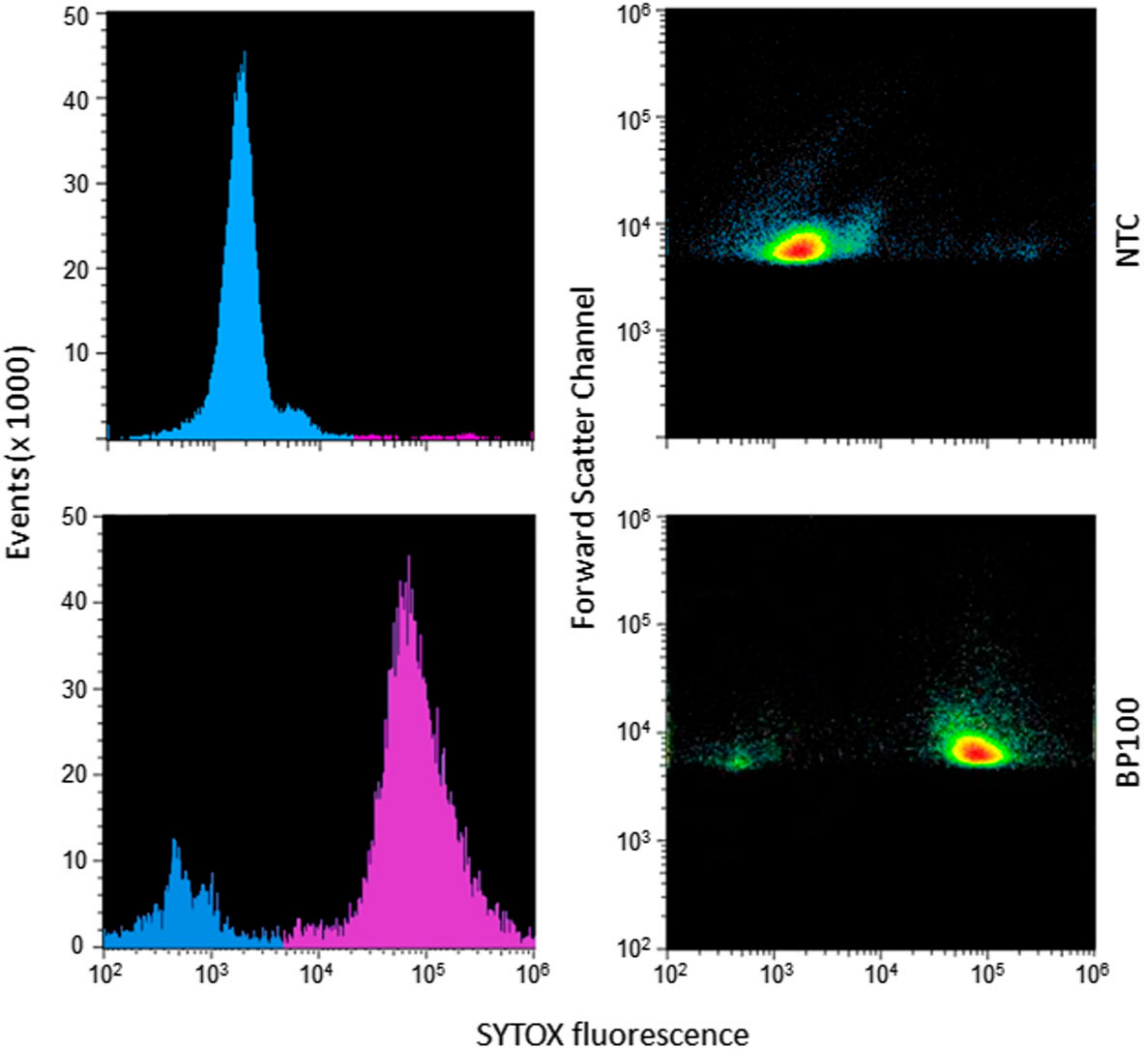
Flow cytometry analysis of *E. amylovora* cells treated with BP100 and stained with SYTOX. Cells suspensions were incubated during 3 h at 25 °C with BP100 at 1.5 μM, and in the presence of SYTOX. A control treated with water was included. *Left panels* represent the number of events while the *right panels* represent the forward scatter channel with SYTOX fluorescence

The effect of BP100 on cell membrane permeability was studied by using SYTOX staining and flow cytometry (Fig. 1). Cells exposed to BP100 were classified into a main group emiting intense fluorescence, and a minor group emiting low fluorescence, whereas unexposed cells showed only very low fluorescence. The intense fluorescence peak was narrow indicating that most cells emitted similar fluorescence intensity. The forward scatter channel showed that the size and morphology of the majority of cells was similar between treated and non-treated cells. Thus, cells treated with BP100 incorporated the SYTOX dye into the cytosol (targetting DNA), but cell morphology and size were not modified compared to non-treated cells.

### Bactericidal activity of BP100 combined with lysozyme

**Fig. 2:**
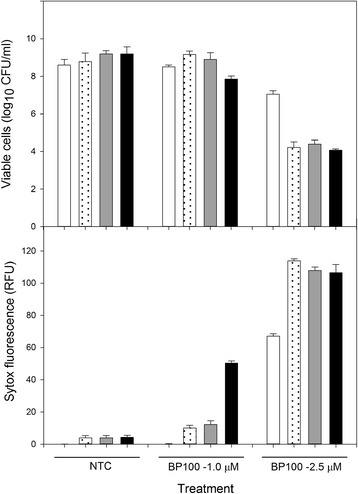
Effect of lysozyme on the activity of BP100 against *E. amylovora*. The activity was determined using viable cells and SYTOX fluorescence of *E. amylovora* suspensions (10^8^ CFU/ml) treated during 1 h with water or BP100 at 1.0 and 2.5 μM, combined with lysozyme at 0 (□), 0.125 (□), 0.250 (*gray square*) and 0.5 (■) mg/ml

**Fig. 3:**
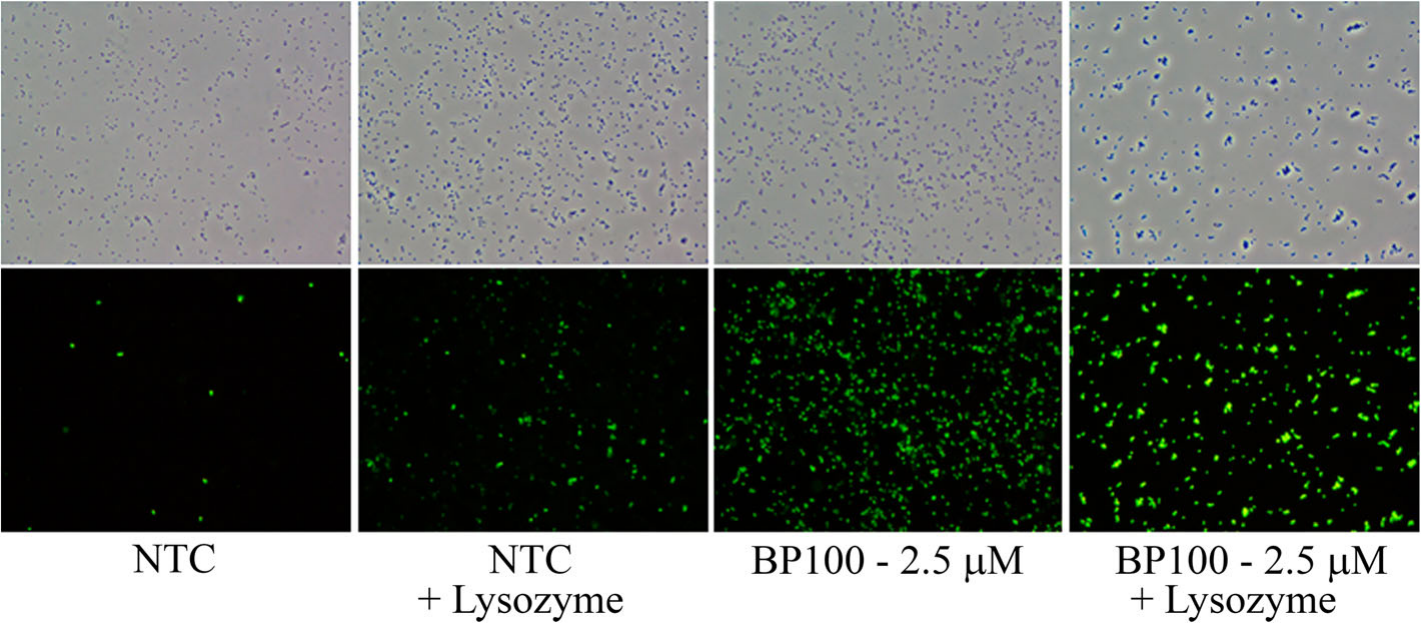
Fluorecense microscope images of *E. amylovora* cells stained with SYTOX. *E. amylovora* cells suspensions (10^8^ CFU/ml) were treated during 1 h with water or BP100 to 2.5 μM, in the presence or absence of lysozyme. Images were taken with an optical microscope using white light (*upper panel*) and ultraviolet light (*lower panel*)

The effect of the combination of BP100 with lysozyme was determined using a contact assay where cell viability and membrane permeation were determined simultaneously (Fig. 2). It can be observed that BP100 or lysozyme alone at the concentrations tested were not active. At 1.0 μM BP100, the effect of the addition of lysozyme was only observed at 0.5 mg/ml (*F* = 9.2; *p* < 0.002), while at 2.5 μM the effect on the growth inhibition was significant at all lysozyme concentrations (*F* = 253.1; *p* < 0.0001). As well as for cell viability similar results were observed for SYTOX fluorescence. Non treated cells presented the normal morphology and absence of fluorescence, while cells treated with lysozyme showed an altered morphology and a baseline fluorescence level (Fig. 3). However, cells treated only with BP100 at 2.5 μM maintained normal morphology but with a strong fluorescence. Cells treated with the combination of BP100 and lysozyme presented strong fluorescence with altered morphology adopting spherical appearance, and a tendency to form aggregates.

**Fig. 4:**
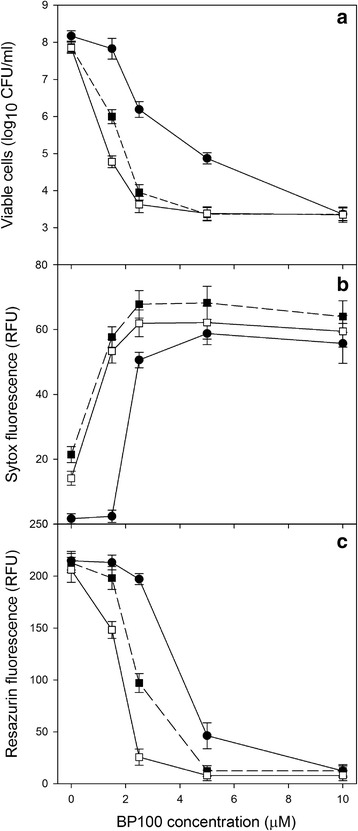
Dose-effect of BP100 alone or combined with lysozyme in the antibacterial activity against *E. amylovora*. The effect was measured as viable cells (**a**), SYTOX green fluorescence (**b**) and resazurin fluorescence (**c**) of *E. amylovora* suspensions (10^8^ CFU/ml). Treatments consisted of 1 h with BP100 at 0, 1.0, 2.5, 5.0 and 10.0 μM, combined with lysozyme at 0 (●), 0,05 (■), 0,50 (□) mg/ml

The effect of BP100 alone or combined with lysozyme was also tested using a contact assay where viable cells, cell membrane damage and metabolic activity were determined simultaneously (Fig. 4). When the concentration of BP100 increased, the viable cells decreased exponentially (from 10^8^ CFU/ml without BP100 to less than 10^4^ CFU/ml at 10 μM). This decrease was clearly enhanced by lysozyme with a reduction of viable cells of 1.5 to 2.0 logs. The effect was more evident at the lower doses of BP100. SYTOX and resazurin measurements showed also similar effects. Thus, SYTOX fluorescence increased when BP100 was combined with lysozyme, while resazurin fluorescence decreased, in both cases indicating membrane damage that simultaneously decrease the metabolic activity of cells.

**Fig. 5:**
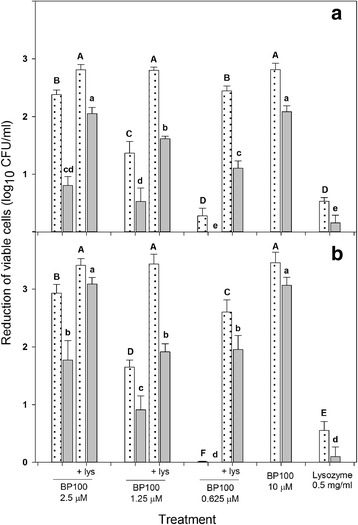
Influence of leaf extract on the antibacterial activity of BP100 alone or combined with lysozyme. The effect was measured as the growth inhibition of *E. amylovora* suspension in each treatment respect the nontreated control after exposure to 2.5, 1.25 and 0.625 μM peptide concentrations without lysozyme or with lysozyme 0.50 mg/ml. The peptide assay was carried out in water (□) or in pear leaf extracts diluted at 10% (gray square). Two independent experiments, assay 1 (**a**) and assay 2 (**b**), were performed. The confidence intervals for the means are indicated on top of the bars. Letters over the bars indicate the significance of the difference between treatment extracts (*P* ≤ 0.05), according to Waller-Duncan’s test

The interference of leaf extracts on the activity of BP100, alone or combined with lysozyme, was also studied (Fig. 5). In this assay, different doses of BP100 (0.625, 1.25, 2.5 and 10 μM), alone or combined with lysozyme at 0.5 mg/ml, were used. The activity of BP100 on the reduction of cell viability was decreased significantly by the presence of leaf extracts (*F* = 134.4, *p* < 0.001). For example, in the first experiment, the reduction of viable cells by BP100 treatment was of 2.38 log in water, while only of 0.81 log in the presence of leaf extracts. The reduction was variable among treatments, but in all the cases the inhibition of *E. amylovora* was reduced in the presence of leaf extracts. However, the combination of BP100 with lysozyme recovered completely or partially the effect of BP100. Interestingly, the effect of the combination of BP100 at 0.625 μM with lysozyme was similar to the treatment with BP100 alone at 2.5 μM. Lysozyme alone showed no significant activity. These results were consistent in both experiments.

### Inhibition of leaf infection by BP100 combined with lysozyme

**Fig. 6:**
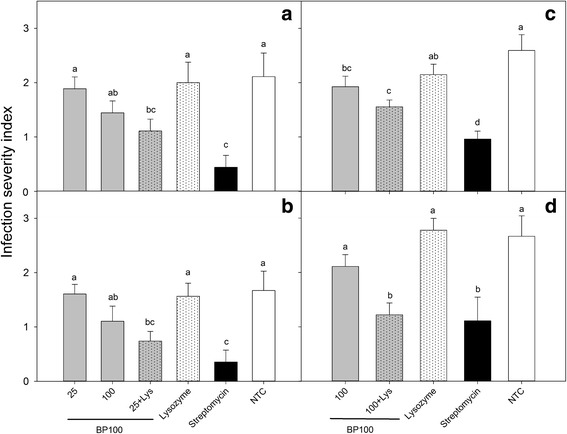
Effect of the preventive application of BP100 and lysozyme in the control of *E. amylovora* infections on pear leaves. BP100 was applied at different concentrations, alone or combined with lysozyme, in detached pear leaves, just 1 h before the inoculation with a suspension of *E. amylovora* (10^8^ CFU/ml). Nontreated control (NTC), lysozyme control and reference treatment with streptomycin were included. Four independent experiments (**a**, **b**, **c** and **d**) were performed. The confidence intervals for the means are indicated on top of the bars. Different letters over a bar indicate a significant difference from the nontreated control for a given experiment, according to Waller-Duncan's test (*P* < 0.05)

**Fig. 7:**
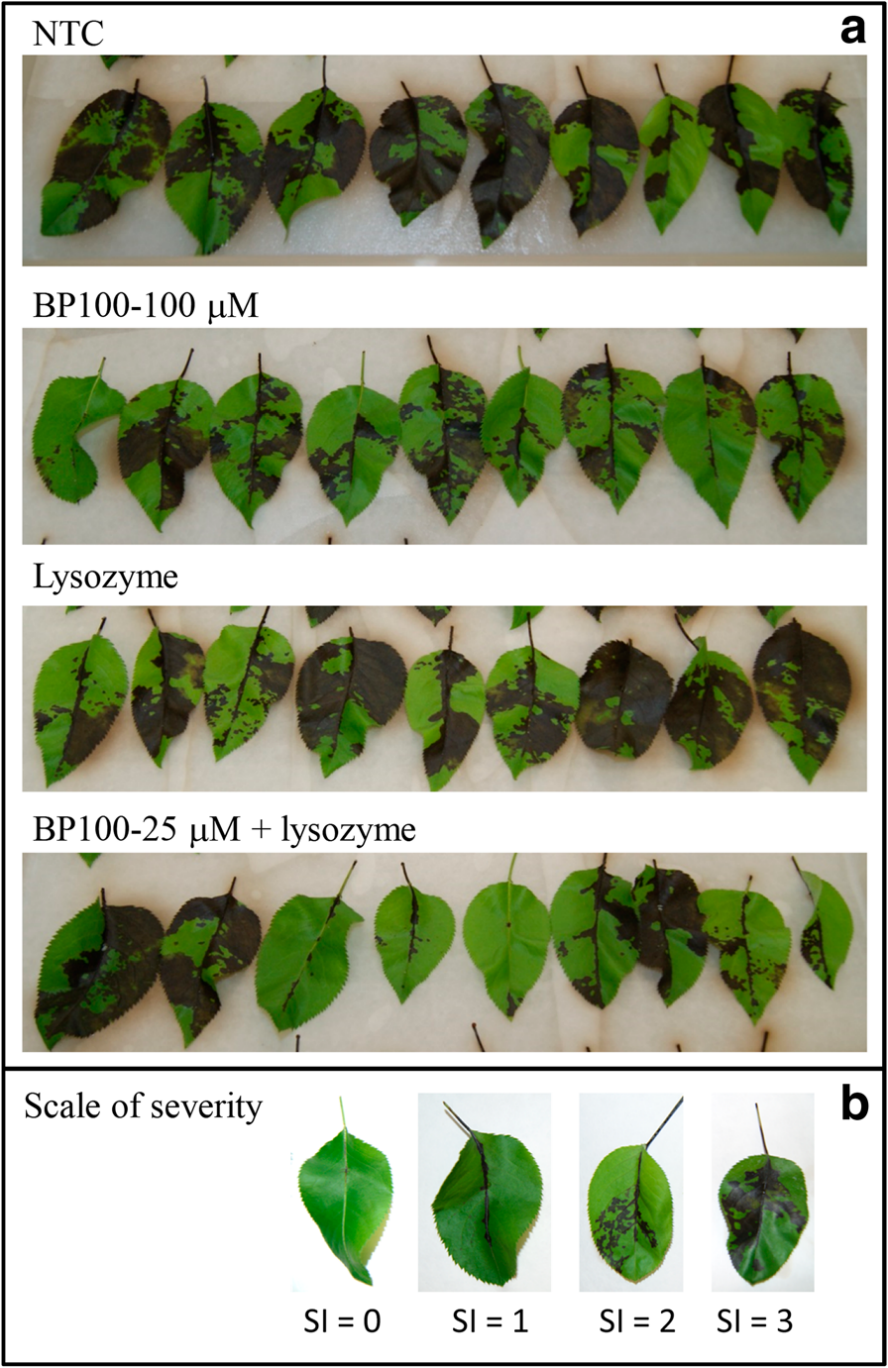
Fire blight symptoms in pear leaves submitted to different treatments with lysozyme and BP100. **a** Wounded leaves were treated with 10 μl of either, water (NTC), BP100 at 100 μM, lysozyme, or BP100 at 25 μM combined with lysozyme. Then, wounds were inoculated with 10 μl of a suspension of *E. amylovora* EPS101 at 10^7^ UFC/ml. Assessment of symptoms was performed after 5 days of incubation at 23 °C under high relative humidity. **b** Scale of severity of infections according to the symptoms observed: 0, no symptoms; 1, leaf necrosis localized around the wound; 2, necrosis progression far from the wound; and 3, necrosis extended to most part of the whole leaf

The effect of BP100 applied preventively, alone or combined with lysozyme, on the inhibition of infections of *E. amylovora* in pear leaves was determined in four assays (Figs. 6 and 7). In the first and second experiment, results were similar and the severity of infections in the non-treated control were 2.1 and 1.7, respectively. In both experiments, only the treatments based on streptomycin and BP100 at 25 μM combined with lysozyme, showed significant decrease in severity with respect to the non-treated control (*F* = 18.2; *p* < 0.0001 and *F* = 16.7, *p* < 0.0001). Treatments based on lysozyme and BP100 alone did not show differences with the non-treated control.

Similar results were observed in the third and fourth experiments though the severity in the non-treated controls was higher than in the previous assays, with values of 2.7 and 2.6, respectively. Significant differences were also observed between BP100 at 100 μM combined with lysozyme and the non-treated control (*F* = 36.9; *p* < 0.0001 and *F* = 25.2, *p* < 0.0001). Lysozyme alone did not show reduction in disease severity, and BP100 alone only presented significant differences with the non-treated control in the third experiment.

Globally, BP100 alone did not have any effect at 25 μM compared to non-treated controls, but it was effective at 100 μM in one out of four experiments. Lysozyme alone had no significant effect in the four assays compared to the non-treated control. However, the efficacy of BP100 at either 25 or 100 μM was significantly enhanced by lysozyme in all four assays. Interestingly, in one assay the effect of BP100 combined with lysozyme did not differ significantly from streptomycin.

## Discussion

In the present work we have shown that the bactericidal effect of BP100 is enhanced by lysozyme. Our results confirm that the bactericidal effect of BP100 against *E. amylovora* is mainly associated to the disruption of the bacterial cell membranes in agreement with some studies performed with BP100 in artificial membranes [36, 37, 50]. Some of these studies conclude that BP100 operates via a carpet mechanism, where peptide penetrates into the hydrophobic core of the bilayer producing a membrane alteration and consequently changing the membrane permeability [51]. The same conclusion has been obtained in the case of CecXJ-37 N, a cationic peptide similar to cecropin, demonstrating that the peptide induced pore-formation, morphological changes and lysed *E. coli* cells [52]. In addition, other authors reported the capacity of a cecropin A-magainin hybride peptide to destroy the integrity of the bacterial cell membrane [53].

In relation with the increase of the activity of BP100 when combined with lysozyme, similar results were obtained in some works showing that the combination of the bacteriocin nisin with lysozyme had a synergistic effect against Gram positive bacteria [32–34] or that the combination of lysozyme with the GMAP2 peptide enhances the activity against several Gram negative bacteria like *Escherichia coli*, *Klebsiella pneumoniae*, *Pseudomonas aeruginosa* and *Salmonella typhimurium* [35]. Interestingly, other studies reported that the combination of nisin and lysozyme caused a rapid depolarization of the cytoplasmic membranes of *Staphylococcus aureus* [31].

The increase of the bactericidal effect of BP100 combined with lysozyme was due to the irreversible cell membrane damage causing a decrease in metabolic activity (resazurin-redution) and cell death. With this combination, MIC was reduced four times compared to BP100 alone and cell death rate increased. The enhacement of the bactericidal activity when combined BP100 with lysozyme, can be explained because of the potential affinity of BP100 with the murein or with some anionic components present in the outer membrane like lipoproteins [54] or lipopolysaccharides [55], that probably reduce the available molecules of BP100 that can interact with the correct target, in this case the inner cell membrane. Thus, the fact that lysozyme hydrolyses murein [56], could favor that BP100 molecules don’t interact with these cell wall components and could better interact with the inner cell membrane. This hypothesis could explain that lower concentrations of BP100 combined with lysozyme presented similar activity than BP100 alone four times concentrated. These results are in agreement with the report that antimicrobial activity of peptide parasin I was enhanced by lysozyme [57]. The authors suggest that lysozyme could allow the access of parasin I to the cytoplasmic membrane thanks to the lysis of peptidoglycan. Moreover, microscopy studies performed in the present work also reinforce this hypothesis, because the combination of BP100 with lysozyme produced spherical cell morphologies and cell aggregates. Spherical cell morphology in several Gram negative bacteria caused by lysozyme has been previously reported in the case of *E. coli* [58] or by combination of lysozyme with high pressure treatments [59]. In addition, the formation of cell aggregates has been reported by treatment with antimicrobial peptides in early stages before the lysis of the cells [60, 61]. The above reports support the hypothesis that the effect of both, BP100 and lysozyme, may be mainly associated to an alteration of the cell envelope.

A decrease of the bactericidal activity of BP100 against *E. amylovora* was observed in plant extracts compared to water solutions, in agreement with other reports for the antimicrobial peptides cecropin B and SB-37 [62], Pep11 and Pep20 [28], Pep3 [27] and several CECMEL11 peptides [26]. However, the combination of BP100 peptide with lysozyme increased its activity in the presence of leaf extracts. This effect was confirmed by leaf infection assays in which the efficacy of BP100 was significantly enhanced by lysozyme, and in one case the effect did not differ from the antibiotic streptomycin. Interestingly, the concentration of BP100 at 25 μM in the presence of lysozyme provided similar efficacy of control than the concentrations of 100–200 μM previously reported when BP100 is applied alone [26].

## Conclusions

The combination of BP100 peptide with lyzozyme increases the bactericidal activity of BP100 against *E. amylovora*, even in the presence of plant extracts, and enhances the protection against fire blight infections in plant material. These results provide the basis for a better formulation of antimicrobial peptides for plant protection.

## Abbreviations

AMP: Antimicrobial peptide
ANOVA: Analysis of variance
CFU: Colony forming units
ESI-MS: Electrospray ionization mass spectrometry
HPLC: High performance liquid chromatography
LB: Lysogenic broth
MALDI-TOF: Matrix-assisted laser desorption/ionization Time-Of-Flight
MIC: Minimal inhibitory concentration
